# Supplementary material for: Targeting the glycan of receptor binding domain with jacalin as a novel approach to develop a treatment against COVID-19
Source: R Soc Open Sci. 2020 Sep 23;7(9):200844. doi: 10.1098/rsos.200844 (PMC7540766; doi:10.1098/rsos.200844)
Supplement: Figures S1-S4 [file rsos200844supp1.doc]

**Targeting the glycan of receptor binding domain with jacalin as a novel approach to develop a treatment against COVID-19**

Senthilnathan Rajendaran, Arunchalam Jothi, Veerappan Anbazhagan*

School of Chemical & Biotechnology, SASTRA Deemed University, Thanjavur – 613 413, Tamil Nadu, India


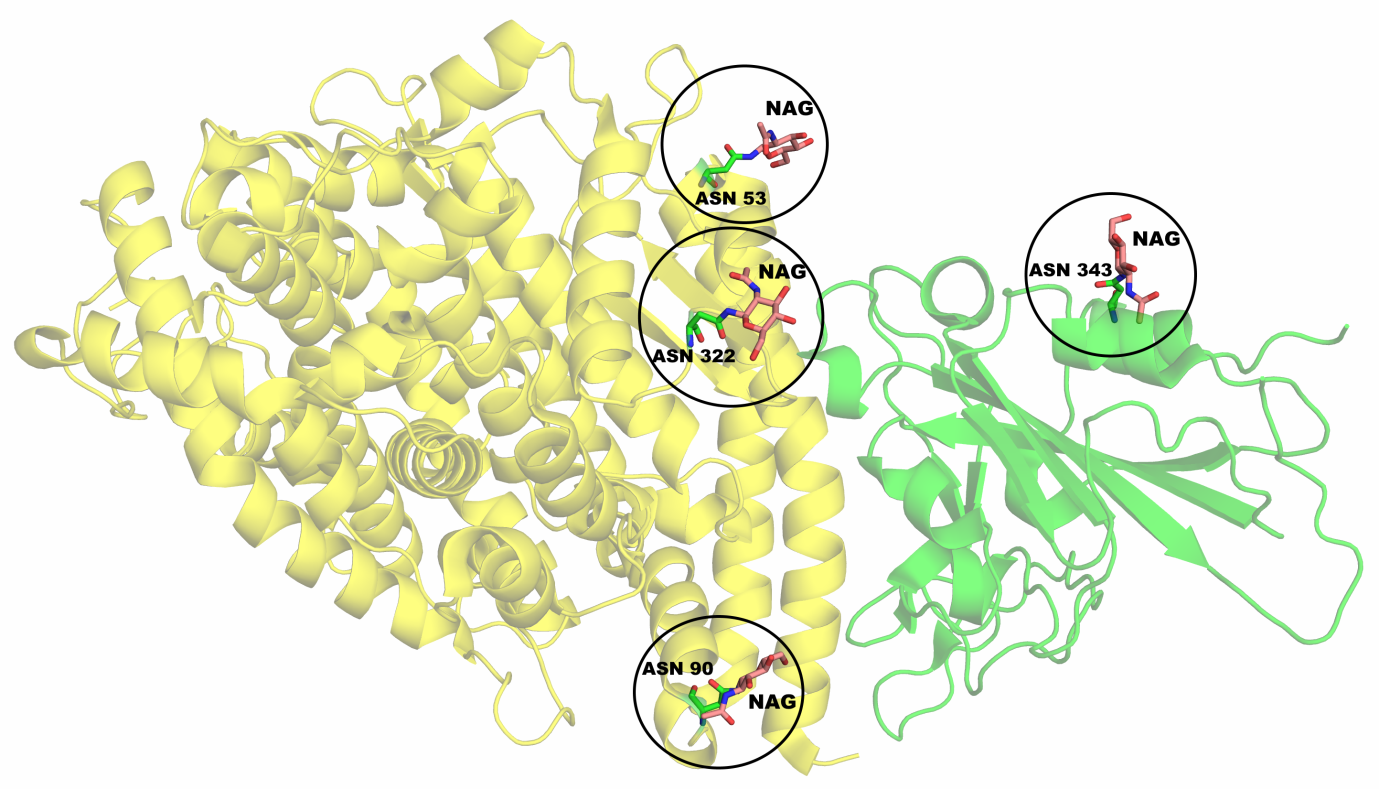


Figure S1: Crystal structure of novel corona spike RBD complexed with ACE2 receptor (PDB ID: 6LZG). Spike RBD shown in green colour, human ACE2 receptor shown in yellow colour and sugar binding glycan sites considered for our analysis are encircled in the figure (NAG: N-Acetyl Glucosamine).


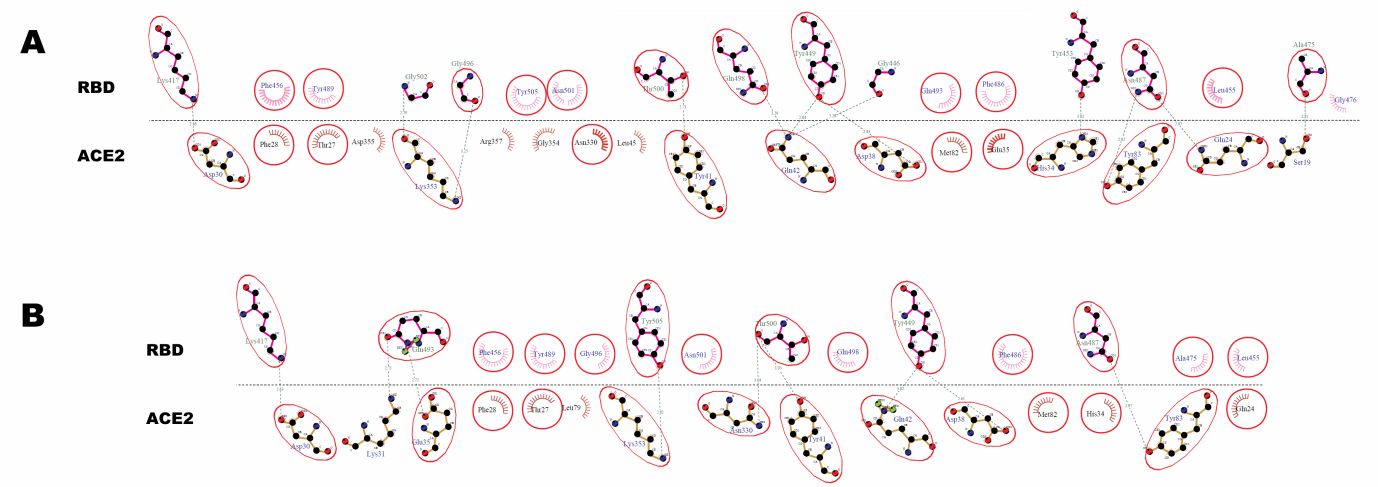


Figure S2: Ligplot diagram showing the interaction between hACE2 and RBD protein structures. (A) From crystal structure complex (PDB Id: 6LZG); (B) From HADDOCK top model structure complex. Similar residues are involved in A and B complexes are encircled. The residues involved in the interaction found by docking procedure was as same as PDB crystal structure, suggests that HADDOCK method is useful to study protein-protein interaction.

Table S1: Analysis of interaction between RBD and jacalin.

| Amino acid residue interactions | | Type of interactions | Distance (Å) |
| --- | --- | --- | --- |
| RBD (A) | Jacalin (B) |
| G339 | F47 | Amide-Pi Stacked | 4.2 |
| L335 | A3 | Alkyl | 4.9 |
| E340 | K2 | Electrostatic | 5.0 |

Table S2: Analysis of van der waal interaction between RBD and jacalin.

| Amino acid residue interactions | | | | Distance (Å) |
| --- | --- | --- | --- | --- |
| RBD (A) | Atoms involved | Jacalin (B) | Atoms involved |
| T333 | CB | D5 | CG | 4.0 |
| L335 | CB | A3 | CB | 3.6 |
| G339 | C | F47 | CZ | 3.5 |
| E340 | CG | F47 | CZ | 3.8 |
| N343 | OD1 | Y78 | CE2 | 3.6 |
| N343 | ND2 | G121 | C | 3.6 |
| N343 | OD1 | Y122 | CB | 3.3 |
| N343 | ND2 | D125 | CG | 3.9 |
| A344 | C | Y122 | CE1 | 3.8 |
| F338 | C | Y1 | C | 3.8 |
| P337 | CB | K2 | CG | 3.9 |


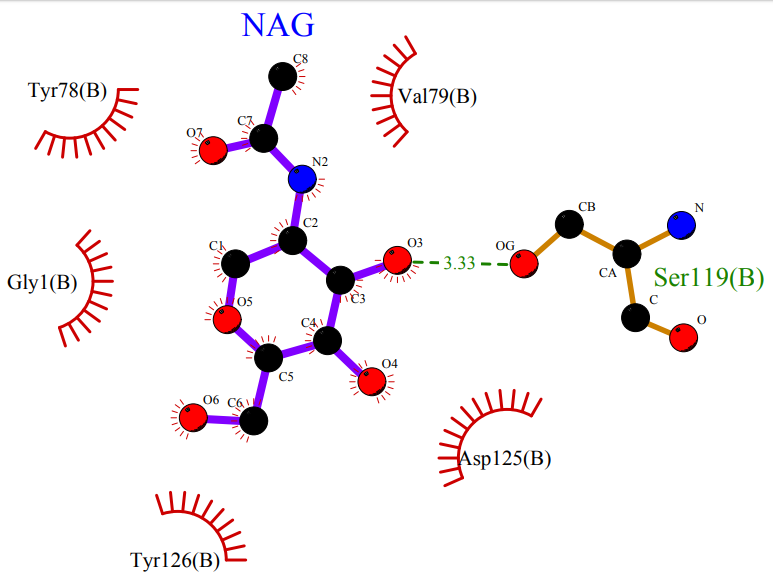


Figure S3: Ligplot depicting the interaction between RBD, NAG of N343 and jacalin.

Table S3: Interaction between RBD and hACE2 in the absence and presence of jacalin.

| Type of Interactions | RBD - hACE2 complex | RBD - hACE2 complex in the presence of jacalin |
| --- | --- | --- |
| H-Bond | 12 | 9 |
| Hydrophobic (Pi-Pi Stack) | 2 | 1 |
| Electrostatic (Salt bridge) | 1 | 1 |
| Van der waals | 13 | 12 |

Figure S4: Ligplot diagram showing the amino acids involved in the interaction between hACE2 and RBD (A) in the absence and (B) in the presence of jacalin. Common amino acid residues involved in the both the conditions are encircled. It is clear from the Figure S3 and Table S3, the binding of jacalin to RBD affects the interactions between RBD and hACE2
